# The conduction velocity-potassium relationship in the heart is modulated by sodium and calcium

**DOI:** 10.1007/s00424-021-02537-y

**Published:** 2021-03-04

**Authors:** D. Ryan King, Michael Entz, Grace A. Blair, Ian Crandell, Alexandra L. Hanlon, Joyce Lin, Gregory S. Hoeker, Steven Poelzing

**Affiliations:** 1 Translational Biology, Medicine, and Health Graduate Program, Virginia Polytechnic Institute and State University, Blacksburg, VA USA; 2 Center for Heart and Reparative Medicine Research, Fralin Biomedical Research Institute at Virginia Tech Carilion, Roanoke, VA USA; 3 Department of Biomedical Engineering and Mechanics, Virginia Polytechnic Institute and State University, Blacksburg, VA USA; 4 Center for Biostatistics and Health Data Science, Virginia Polytechnic Institute and State University, Roanoke, VA USA; 5 Department of Mathematics, California Polytechnic State University, San Luis Obispo, CA USA; 6 School of Medicine, Virginia Tech Carilion, Roanoke, VA USA

**Keywords:** Hyperkalemia, Sodium, Calcium, Cardiac Electrophysiology, Langendorff

## Abstract

The relationship between cardiac conduction velocity (CV) and extracellular potassium (K^+^) is biphasic, with modest hyperkalemia increasing CV and severe hyperkalemia slowing CV. Recent studies from our group suggest that elevating extracellular sodium (Na^+^) and calcium (Ca^2+^) can enhance CV by an extracellular pathway parallel to gap junctional coupling (GJC) called ephaptic coupling that can occur in the gap junction adjacent perinexus. However, it remains unknown whether these same interventions modulate CV as a function of K^+^. We hypothesize that Na^+^, Ca^2+^, and GJC can attenuate conduction slowing consequent to severe hyperkalemia. Elevating Ca^2+^ from 1.25 to 2.00 mM significantly narrowed perinexal width measured by transmission electron microscopy. Optically mapped, Langendorff-perfused guinea pig hearts perfused with increasing K^+^ revealed the expected biphasic CV-K^+^ relationship during perfusion with different Na^+^ and Ca^2+^ concentrations. Neither elevating Na^+^ nor Ca^2+^ alone consistently modulated the positive slope of CV-K^+^ or conduction slowing at 10-mM K^+^; however, combined Na^+^ and Ca^2+^ elevation significantly mitigated conduction slowing at 10-mM K^+^. Pharmacologic GJC inhibition with 30-μM carbenoxolone slowed CV without changing the shape of CV-K^+^ curves. A computational model of CV predicted that elevating Na^+^ and narrowing clefts between myocytes, as occur with perinexal narrowing, reduces the positive and negative slopes of the CV-K^+^ relationship but do not support a primary role of GJC or sodium channel conductance. These data demonstrate that combinatorial effects of Na^+^ and Ca^2+^ differentially modulate conduction during hyperkalemia, and enhancing determinants of ephaptic coupling may attenuate conduction changes in a variety of physiologic conditions.

**Supplementary Information:**

The online version contains supplementary material available at 10.1007/s00424-021-02537-y.

## Introduction

Coordinated electrical activity in the heart is facilitated through a sequence of transmembrane ion exchange events governed by channels, pumps, and exchangers [4]. A major determinant of the speed of cell-to-cell electrical action potential propagation (conduction velocity; CV) in cardiac tissue is the inward sodium current (*I*_Na_) [8, 4]. It is well appreciated that *I*_Na_ is modified by changes in the resting membrane potential (RMP) of the cardiomyocyte [32, 74]. During diastole, RMP is primarily maintained by potassium currents, [33, 31] and changing extracellular potassium (K^+^) concentration will directly alter the RMP. Depending on RMP, sodium (Na^+^) channel availability and *I*_Na_ can also be modulated.

Given the dense expression of the voltage gated sodium channel Nav1.5 in the intercalated disc [55] and more specifically in the gap junction (GJ) adjacent perinexus [57, 70], recent evidence suggests that reducing *I*_Na_ exacerbates conduction slowing when intercellular separation in the perinexus is widened by altering an electrical field coupling (i.e., ephaptic coupling, EpC) pathway parallel to gap junctional coupling (GJC). In brief, EpC posits that extracellular potential perturbations in shared clefts between cells can transactivate apposing voltage-gated ion channels. Computational models demonstrate EpC can be modified by changing the cleft width between neighboring cardiomyocytes, particularly in the GJ adjacent perinexus [58, 23], and by altering ionic concentrations within intercellular clefts [63, 38, 48, 40, 66, 73]. However, it remains unknown how EpC may modulate the relationship between CV and extracellular K^+^.

In guinea pigs, both experimental and computational models demonstrate that the CV-K^+^ relationship is biphasic. [32, 52, 35] CV is positively correlated with K^+^ until approximately 8 mM by a mechanism of reducing the potential difference between RMP and Na^+^ channel activation threshold (i.e., increasing excitability) [32, 61]. Once K^+^ increases to the point that RMP exceeds Na^+^ channel activation threshold, CV will subsequently decrease as the number of Na^+^ channels in the inactive state increases [25, 74].

It is important to note here that GJC, and not EpC, is still considered the principal mediator of electrical propagation between cardiomyocytes. This is important because GJC may also modulate the relationship between CV and K^+^. Though, the historic relationship between CV and GJC, particularly mediated by the principal ventricular GJ protein connexin 43 (Cx43), is not straightforward. While GJ functional expression should correlate with CV [10], our previous work documented the myriad of CV responses associated with the Cx43 heterozygous knockout mouse and demonstrated that electrolyte composition in crystalloid perfusates can significantly modulate CV slowing in hearts with genetically reduced Cx43 expression [18, 14, 17]. The mechanism by which GJ-dependent CV can be modulated by electrolyte composition is via EpC, because GJC alters intracellular potentials while EpC modulates both extracellular potentials and extracellular ion concentrations. Together, dynamic changes in transmembrane and reversal potentials within intercalated disc nanodomains interact to rapidly propagate the action potential wave front, while simultaneously acting in a negative feedback mechanism (self-attenuation) to produce often invariant conduction until one form of coupling is altered dramatically enough to overwhelm the other mechanism and slow conduction [19].

Recent evidence supports EpC as a mechanism that modulates cardiac electrophysiology in response to GJ uncoupling [68, 18, 11, 14], reduced inward rectifier potassium current [71], reduced sodium channel beta subunit adhesion [69], sodium channel gain-of-function [19, 50], atrial fibrillation [56], inflammatory cytokines [15, 45], and ischemia [16, 27]. With regard to this last case, ischemia is often associated with elevated K^+^, leading us to hypothesize that the CV-K^+^ relationship can also be altered via electrolyte modification. More specifically, we hypothesize that increasing perfusate Na^+^ will increase the sodium reversal potential and therefore peak *I*_Na_, and increasing calcium (Ca^2+^) will, among other things, narrow or attenuate perinexal expansion [14, 16, 27]. This study also tests whether GJC is a modulator of the CV-K^+^ relationship in the context of altered Na^+^ and Ca^2+^. Our data demonstrate that combinatorial effects of Na^+^ and Ca^2+^ differentially preserve CV during hyperkalemia and suggest that enhancing determinants of EpC may attenuate CV changes in a variety of physiologically relevant conditions.

## Methods

All studies were designed to adhere to the guidelines set forth by the Institutional Animal Care and Use Committee at Virginia Polytechnic Institute and State University and NIH *Guide for the Care and Usage of Laboratory Animals*.

### Langendorff perfusion

Adult male Hartley albino guinea pigs (Hilltop, Scottdale, PA, *n* = 85, 800–1,200 g, 14–16 months old) were anesthetized using isofluorane (4% in O_2_). After loss of peripheral stimuli response, the heart was excised and rapidly cannulated (<4 minutes) for retrograde perfusion in a 3-D printed PLA bath [12]. The lab standard crystalloid perfusate contained (in mM) 140 NaCl, 5.0 NaOH, 4.56 KCl, 1.25 CaCl_2_•2H_2_O, 5.5 dextrose, 0.7 MgCl_2_•6H_2_O, and 10 HEPES. The perfusate was equilibrated to a pH of 7.4 using NaOH or HCl, as necessary, at 37.0°C. Perfusion occurred at a constant flow to maintain coronary pressure between 40 and 60 mmHg. Atria were removed to inhibit competitive stimulation, and ventricles were paced with an AgCl wire placed on the anterior left ventricular epicardium; the ground electrode was placed within the superfusion bath. Stimulation strength was set at 1.5 times the excitation threshold with 5-ms pulse duration and delivered at a basic cycle length of 300 ms.

Using the lab standard perfusate as a baseline, Na^+^, Ca^2+^, and K^+^ were varied. Specifically, Na^+^ was changed from 145 to 155 mM, and Ca^2+^ was changed from 1.25 to 2.0 mM. For each individual experiment, Na^+^ and Ca^+^ were held constant, while K^+^ was varied between four concentrations (4.6, 6.4, 8.0, and 10 mM). In total, there were 16 unique combinations of Na^+^, Ca^2+^, and K^+^ used in this study,

GJs were inhibited by perfusion of the nonspecific GJ uncoupler carbenoxolone (CBX, 30 μM), which has previously been shown to decrease CV [20]. For experiments containing CBX, the first measurements were taken after 15 minutes of perfusion, as time control studies indicated CV reached steady state within that timeframe (data not shown). Subsequent measurements were made at 10-minute intervals.

### Electrocardiography

A volume-conducted bath electrocardiogram (ECG) was recorded using AgCl electrodes, collected at 1 kHz. Electrodes were placed on both sides of the ventricles, with the ground placed at the rear of the bath. Asystole was defined as a lack of discernable ECG at any point during perfusion.

### Transmission electron microscopy

**Fig. 1:**
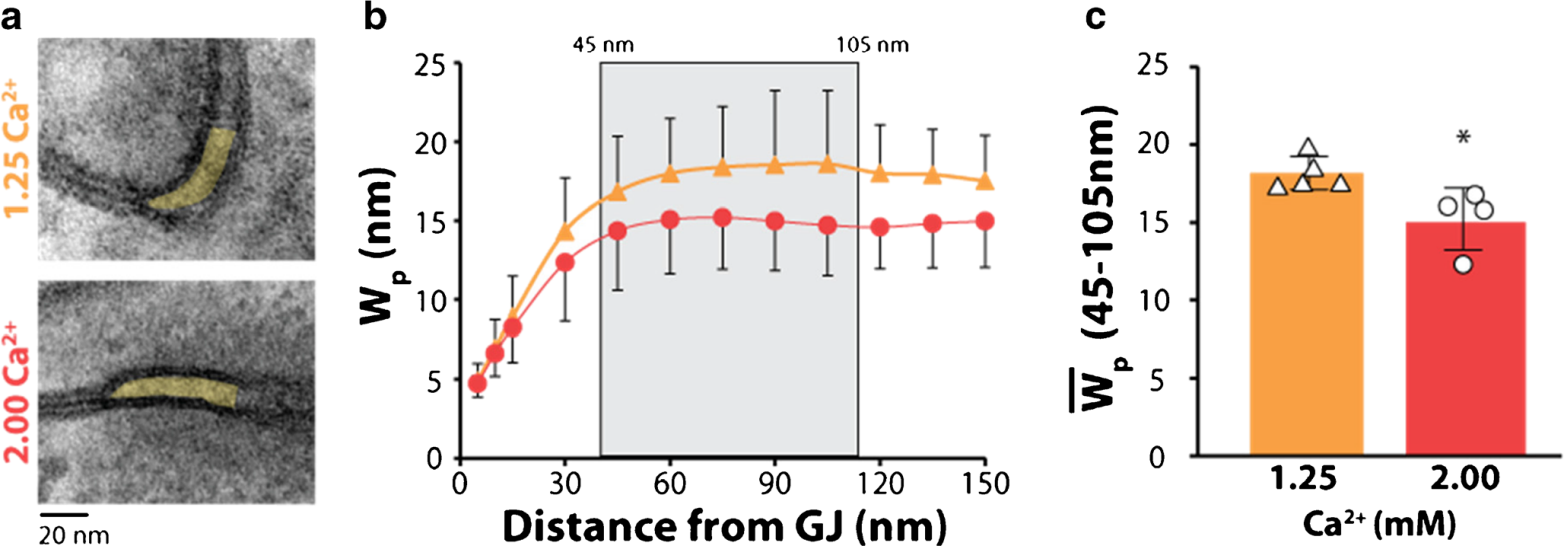
Increasing Ca^2+^ decreases *W*_p_ in the presence of 155-mM Na^+^. (a) Representative transmission electron micrographs of perinexi with 1.25- and 2.00-mM Ca^2+^, (b) *W*_p_ measurements from 0 to 150 nm from GJ plaque, (c) Average *W*_p_ measurements for 45–105 nm from GJ plaque, *W*_p_ significantly decreased with 2.00-mM Ca^2+^. (*n* = 5 hearts perfused with 1.25-mM Ca^+2^ and *n* = 4 hearts with 2.00-mM Ca^2+^, *p < 0.05 compared to 1.25-mM Ca^2+^ via two-tailed Student’s *t* test)

Tissue was sectioned into 1 mm^3^ cubes from the anterior left ventricular free wall (*n* = 5 hearts perfused with solutions containing 1.25-mM Ca^2+^ and *n* = 4 hearts perfused with 2.0-mM Ca^2+^, 3 tissue samples per heart and between 6 and 15 perinexal images per sample). The tissue was fixed overnight in 2.5% glutaraldehyde at 4°C, washed and transferred to PBS, and stored at 4°C. The tissue was processed as previously described [11]. Images of the GJ-adjacent perinexus were collected at 150,000× magnification on a transmission electron microscope (JEOL JEM1400). ImageJ (NIH) was used for manual segmentation of the perinexus. Perinexi were analyzed starting at the point directly adjacent to GJs, measuring up to 150 nm from the GJ plaque as previously described [11]. Changes in perinexal width (*W*_p_) were analyzed as the average intermembrane separation at distances between 45 and 105 nm from the GJ plaque. The average *W*_P_ for a heart was the average *W*_P_ for every perinexi collected from that heart (between 6 and 15 perinexi per heart). Raw datapoints in Fig. 1 are the average *W*_P_ for individual hearts.

### Optical mapping

Following a brief period of stabilization (15 minutes), hearts were perfused with the voltage sensitive dye di-4-ANEPPS (7.5 μM) followed by a 10-minute washout period. The electro-mechanical uncoupler 2,3-butanedione monoxime (BDM, 7.5 mM) was used to decrease cardiac motion. In order to further stabilize the heart for imaging, light mechanical pressure was placed on the posterior surface of the heart. The fluorophore was excited by a halogen light source (MHAB-150 W, Moritex) equipped with a fiber light guide and 520/35-nm band-pass filter (Brightline). The filtered excitation light was directed onto a dichroic mirror (565 nm, Chroma Technology) and reflected onto the heart via epi-illumination. Emitted light was collected via a tandem lens system and transmitted through a 610-nm long-pass filter (Andover Corp.) before detection by a MiCam Ultima L-type CMOS camera (SciMedia: 100 × 100 pixels, field of view—15.9 × 15.9 mm). Baseline optical action potentials were recorded at a 1-kHz sample rate for a duration of ~2 seconds during intrinsic activity and steady-state pacing at a 300-ms cycle length.

Cardiac CV was calculated as previously described [6, 16]. Briefly, activation time for each pixel was determined as the maximum rate of optical action potential rise. CV was quantified in two directions, longitudinal (CV_L_) and transverse (CV_T_). Conduction in each direction was quantified by selecting vectors within five pixels and an angle of ±8° from a user-defined direction of longitudinal (fastest) and transverse (slowest) propagation. Conduction vectors immediately adjacent to the pacing site were excluded to reduce pacing artifacts. Data are presented herein as representative isochrone maps with 3-ms time steps.

### Statistical analysis

Statistical analyses were performed in GraphPad Prism 7 and R version 3.6.0. Within R, mixed effect models were fit using the Ime4 library [5]. For all data, *p* < 0.05 was considered statistically significant. Data from a total of 85 hearts are reported in this study. Specific *n* values for each experimental group are included in the figure legends. All summary data are presented as mean ± standard error unless otherwise noted. Details of specific statistical analyses used are included in figure legends.

Experiments were performed in a blinded and randomized fashion. The experimentalist was blinded to the Na^+^, Ca^2+^, and K^+^ concentrations of all perfusion fluids. Likewise, the order of the blinded perfusion solutions was randomized such that neither the experimentalist nor data analyst had knowledge of the perfusate contents until the conclusion of all analyses.

### Computational simulations

To compare the experimental data with a computational model, we used equations that incorporated the importance of the junctional cleft, as done previously [40, 41]. Simulations were run using a strand of 50 cells, shaped like rectangular prisms, withan ionic current model that tracks ion concentrations [43]. GJs were located only on the ends of the cells, along with the majority of the fast sodium ion channels. The intracellular and extracellular current equations discretized both the intracellular and extracellular space, described previously [42], were integrated in time using a Crank-Nicolson scheme and integrated in space using centered finite differences. A strand was chosen for computational efficiency and with the prior assumption that the number of cell-to-cell junctions per unit length is a determinant of conduction [70]. Hence, the strand model can inform longitudinal or transverse conduction without confounding variables introduced by 2- and 3-D models such as wave-front curvature.

Current was briefly injected into the strand to initiate an action potential, which traveled down the cable. The times at which cells in the middle of the strand depolarized were recorded to compute CV. The nominal value of GJC was 666 mS/cm^2^, and cellular length and width were 0.01 cm and approximately 0.0017 cm, respectively (6:1 ratio), and membrane capacitance was held at 1 μF/cm^2^. Extracellular space had a conductivity of 20 mS/cm, with the lateral width of 10^−5^ cm and perinexal width of 1.5 × 10^−6^ cm.

## Results

### Perinexal width

We previously demonstrated that elevating perfusate Ca^2+^ can reduce perinexal width (*W*_p_) in hearts perfused with 145-mM Na^+^ [18, 27]. To confirm this finding when hearts are perfused with 155-mM Na^+^, we here present representative transmission electron micrographs of hearts perfused with 155-mM Na^+^ and either 1.25- or 2.00-mM Ca^2+^ (Fig. 1a). The measurements from 45 to 105 nm were averaged for each heart (Fig. 1b) and compared between Ca^2+^ concentrations. Consistent with our previous findings, increasing Ca^2+^ to 2.00 mM can decrease *W*_p_ compared to 1.25-mM Ca^2+^ (Fig. 1c) [18, 27].

### Conduction velocity

**Fig. 2:**
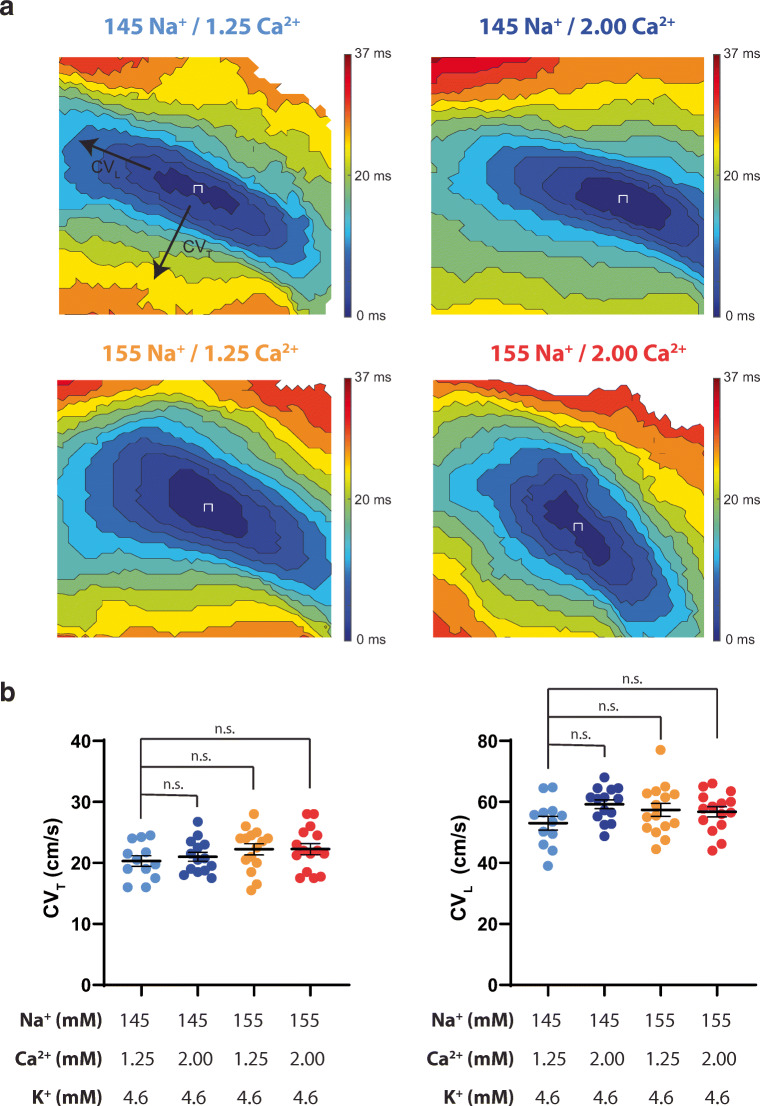
Altering Na^+^, Ca^2+^, or Na^+^ and Ca^2+^ does not change CV_T_ or CV_L_ at 4.6-mM K^+^. (a) Representative isochrone maps for each Na^+^/Ca^2+^ perfusion combination. The 145-mM Na^+^/1.25-mM Ca^2+^ map is marked with CV_T_ and CV_L_ designations for visualization purposes, (b) Summary of CV_T_ and CV_L_ at 4.6-mM K^+^. *p* < 0.05 denoted by *, significance determined by one-way ANOVA with Dunnett’s correction for multiple comparisons. (*n* = 12, 14, 15, 15 from left to right respectively for both CV_T_ and CV_L_)

Representative isochrone maps and summary data of all Na^+^ and Ca^2+^ perfusate combinations at 4.6-mM K^+^ are presented in Fig. 2a. Altering Na^+^ and/or Ca^2+^ at baseline, in the presence of 4.6-mM K^+^, does not significantly change CV_T_ or CV_L_, as can be seen in the summary data within Fig. 2b.

### Transverse conduction velocity

**Fig. 3.**
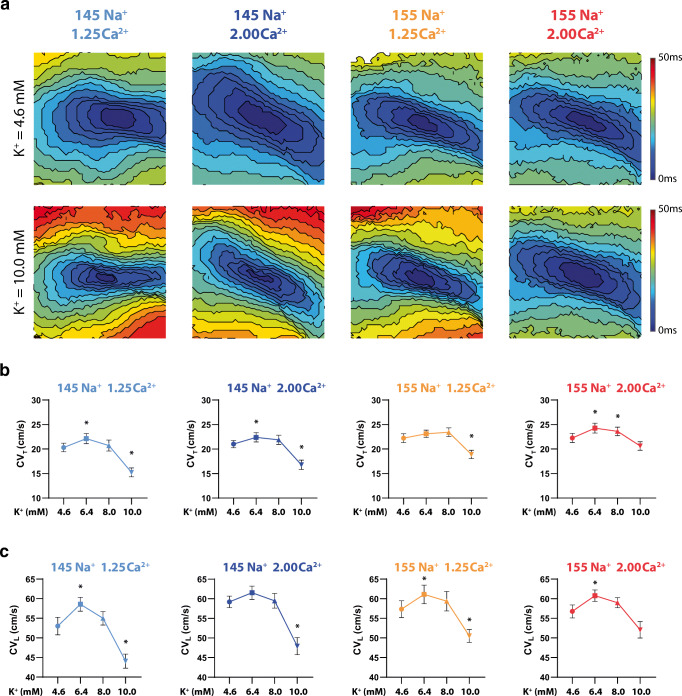
Simultaneously increasing Na^+^ and Ca^2+^ preserves CV_T_ and CV_L_ at 10.0-mM K^+^. (a) Representative isochrone maps for each Na^+^/Ca^2+^ perfusion combination at 4.6- and 10.0-mM K^+^, (b) Summary of CV_T_ as a function of K^+^ for all Na^+^ and Ca^2+^ perfusion combinations, (c) Summary of CV_L_ as a function of K^+^ for all Na^+^ and Ca^2+^ perfusion combinations. *p* < 0.05 denoted by *, significance determined by one-way ANOVA with Dunnett’s correction for multiple comparisons (*n* = 12, 14, 15, 15 from left to right, respectively).

As expected, varying K^+^ between 4.6 and 10.0 mM produces a biphasic response in CV_T_ (Fig. 3; Supplemental Figure 2). Representative isochrone maps in Fig. 3a suggest certain ionic combinations increase CV, as evidenced by fewer isochrones and colors, while other maps suggest conduction slowing. Specifically, faster CV_T_ (supernormal conduction) is observed at 6.4-mM K^+^ in hearts perfused with the 145-mM Na^+^/1.25-mM Ca^2+^, 145-mM Na^+^/2.00-mM Ca^2+^, and 155-mM Na^+^/2.00-mM Ca^2+^ solutions. The 155-mM Na^+^/2.00-mM Ca^2+^ group also demonstrates faster CV_T_ at 8.0-mM K^+^ (Fig. 3b). CV_T_ slowing with severe hyperkalemia (10-mM K^+^) is observed in hearts perfused with three of the four solutions: 145-mM Na^+^/1.25-mM Ca^2+^, 145-mM Na^+^/2.00-mM Ca^2+^, and 155-mM Na^+^/1.25-mM Ca^2+^ solutions (Fig. 3a and b). Yet, CV_T_ is not significantly different at 10-mM K^+^ relative to 4.6-mM K^+^ when hearts are perfused with 155-mM Na^+^/2.00-mM Ca^2+^ (Supplemental Figure 1; Supplemental Table 1). These results suggest that the combination of elevated Na^+^ and elevated Ca^2+^ can attenuate CV_T_ slowing caused by severe hyperkalemia. We further attempted to compare the CV_T_-K^+^ relationship across Na^+^/Ca^2+^ variations by comparing the datasets fit with a quadratic model (Supplemental Figure 2). Unfortunately, this approach did not reveal any statistically significant differences among curves.

### Longitudinal conduction velocity

Similar to the analysis of CV_T_, the relationship of CV_L_ and K^+^ between 4.6 and 10.0 mM is biphasic (Fig. 3c; Supplemental Figure 2). Faster CV_L_ is observed at 6.4-mM K^+^ in hearts perfused with the 145-mM Na^+^/1.25-mM Ca^2+^, 155-mM Na^+^/1.25-mM Ca^2+^, and 155-mM Na^+^/2.00-mM Ca^2+^ solutions (Fig. 3c). CV_L_ slowing with severe hyperkalemia (10-mM K^+^) is observed in hearts perfused with the 145-mM Na^+^/1.25-mM Ca^2+^, 145-mM Na^+^/2.00-mM Ca^2+^, and 155-mM Na^+^/1.25-mM Ca^2+^ solutions (Fig. 3c). Consistent with CV_T_, CV_L_ is not significantly different in hearts perfused with 155-mM Na^+^/2.00-mM Ca^2+^ at 10-mM K^+^ relative to 4.6-mM K^+^. Taken together, these results further support the finding that the combination of elevated Na^+^ and Ca^2+^ can preferentially attenuate CV slowing caused by severe hyperkalemia.

### Incidence of asystole

**Fig. 4:**
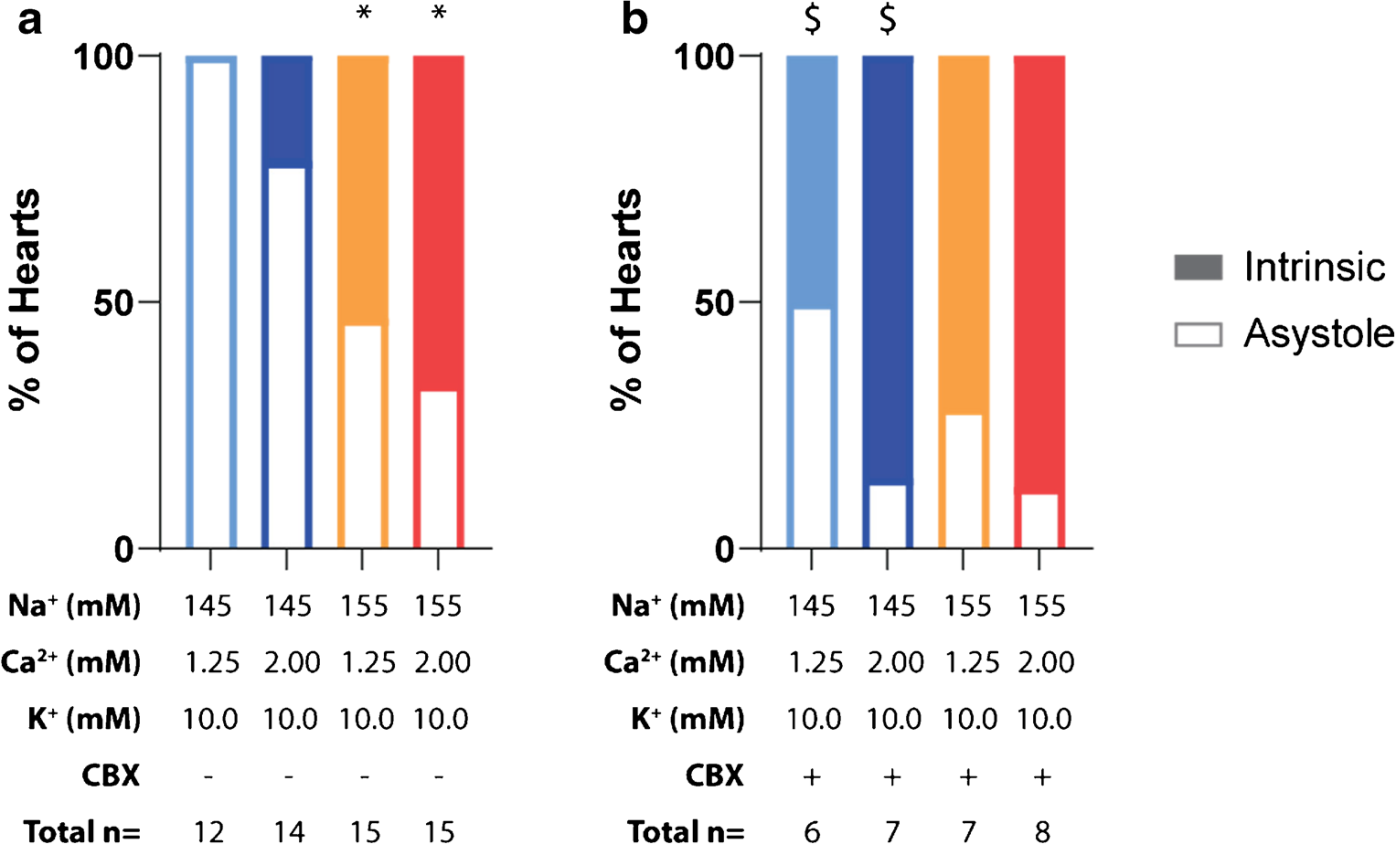
Incidence of asystole during 10.0-mM K^+^ perfusion. (a) Elevating perfusate Na^+^ significantly increases the preservation of intrinsic rhythm at 10.0-mM K^+^ perfusion (significance determined by Fisher’s exact test; * denotes *p* < 0.05 as compared to 145-mM Na^+^, 1.25-mM Ca^2+^, 10.0-mM K^+^ perfusate group), (b) Inhibiting GJs with CBX (30 μM) significantly increases the preservation of intrinsic rhythm in the presence of 145-mM Na^+^ when compared to the control condition (significance determined by Fisher’s exact test; $ denotes *p* < 0.05 compared to each perfusates’ respective CBX – group). There were no significant differences in preservation of intrinsic rhythm across groups perfused with CBX

A surprising finding in the study was that incidence of asystole at 10-mM K^+^ varied among the different ionic concentrations. Specifically, all hearts perfused with the 145-mM Na^+^/1.25-mM Ca^+^ solution develop asystole in the presence of 10-mM K^+^ (Fig. 4a). Elevating Ca^2+^ alone does not significantly reduce the incidence of asystole (145-mM Na^+^/2.00-mM Ca^2+^). However, asystole is significantly reduced in hearts perfused with 155-mM Na^+^ with or without elevated Ca^2+^.

To further probe the mechanism of intrinsic rhythm preservation with elevated Na^+^, we pharmacologically inhibited GJC with CBX (30 μM). Interestingly, CBX significantly reduces the development of asystole at 10-mM K^+^ in the 145-mM Na^+^ perfusion groups but does not further reduce the incidence of asystole in the 155-mM Na^+^ perfusion groups (Fig. 4b).

### Carbenoxolone

**Fig. 5:**
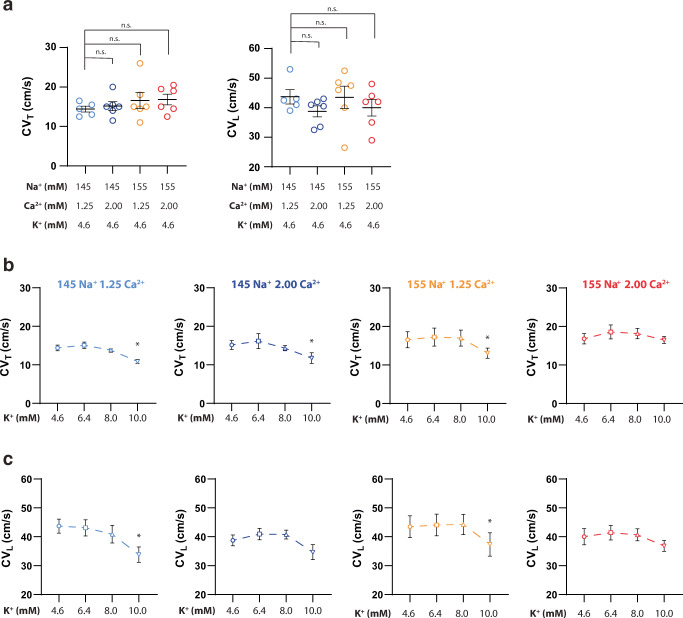
Following GJ inhibition with CBX, simultaneously increasing Na^+^ and Ca^2+^ preserves CV_T_ and CV_L_ at 10.0-mM K^+^. (a) Altering Na^+^, Ca^2+^, or Na^+^ and Ca^2+^ does not change CV_T_ or CV_L_ at 4.6-mM K^+^, (b) Summary of CV_T_ as a function of K^+^ in the presence of CBX (*n* = 6, 7, 7, 8 from left to right, respectively), (c) Summary of CV_L_ as a function of K^+^ in the presence of CBX (*n* = 6, 7, 7, 8 from left to right, respectively). Significance determined by ordinary one-way ANOVA with Dunnett’s correction for multiple comparisons (*p* < 0.05 denoted by *)

We find that CBX reduces CV regardless of Na^+^, Ca^2+^, or K^+^ concentrations, consistent with previous findings [20]. In summary, CV values in Fig. 5a are significantly reduced at 4.6-mM K^+^ with CBX relative to measured values in Fig. 3 (*p* < 0.05 for all comparisons), but once again the combinations of Na^+^ and Ca^2+^ do not produce significant differences in CV_T_ (Fig. 5b) or CV_L_ (Fig. 5c) at 4.6-mM K^+^ with CBX. Interestingly, the expected CV increase between 4.6- and 8-mM K^+^ is not observed with CBX with any Na^+^ or Ca^2+^ combination, and a post hoc comparison of the CV change between 4.6-mM and 6.4-mM K^+^ is also not significantly different for any experimental combination with or without CBX.

Similar to our studies without CBX, CV slowing with severe hyperkalemia (10-mM K^+^) is observed in hearts perfused with 145-mM Na^+^/1.25-mM Ca^2+^ (CV_T_ and CV_L_ slowing), 145-mM Na^+^/2.00-mM Ca^2+^ (CV_T_ slowing only), and 155-mM Na^+^/1.25-mM Ca^2+^ (CV_T_ and CV_L_ slowing) solutions (Fig. 5b and c). Furthermore, CV does not significantly decrease at 10-mM K^+^ with 155-mM Na^+^/2.00-mM Ca^2+^.

### Computational model predictions

**Fig. 6:**
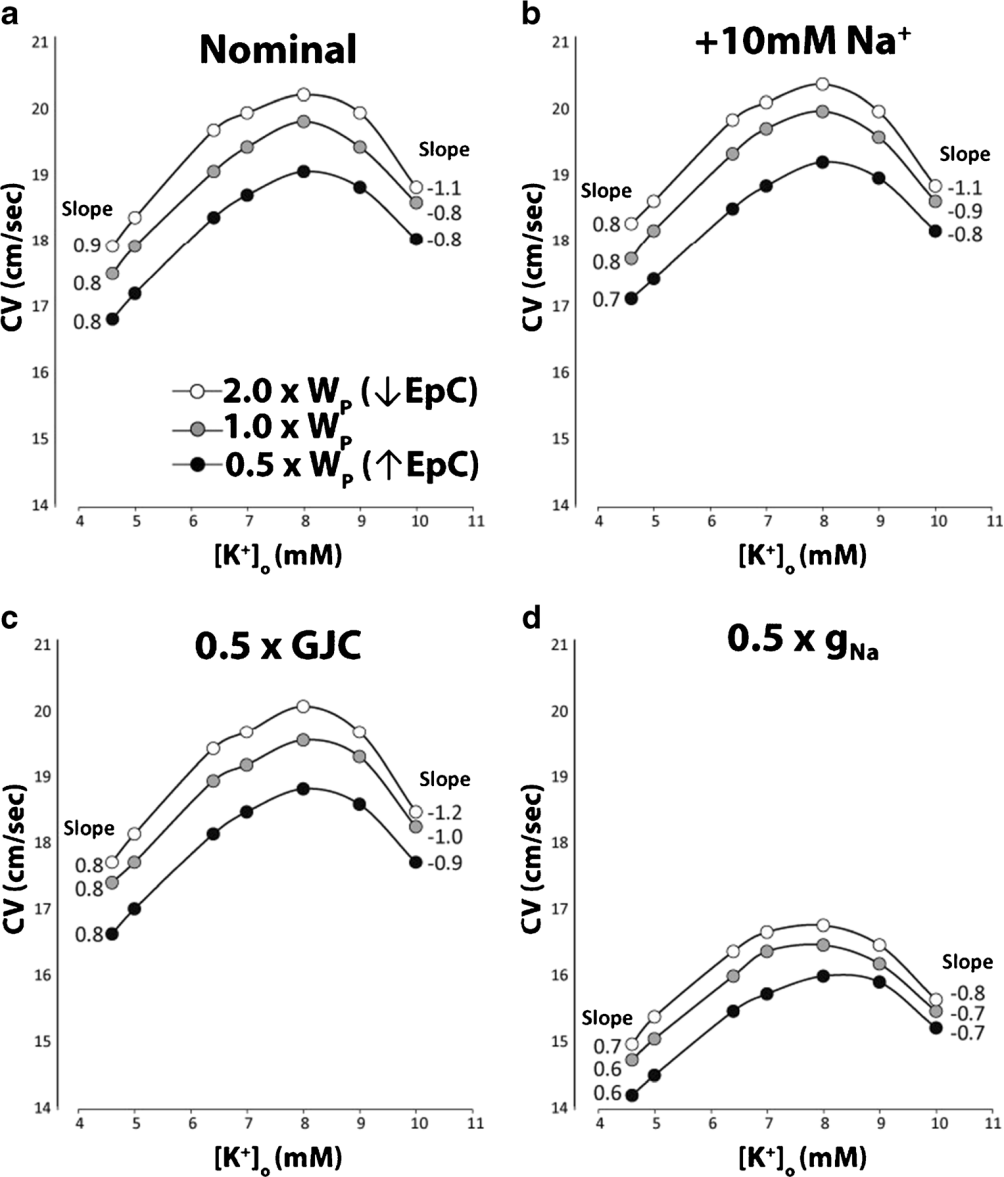
Computational predictions of modulating perinexal width (*W*_P_), extracellular sodium concentrations (Na^+^), gap junctional coupling (GJC), and the fast sodium channel conductance (gNa). (a) Increasing *W*_P_ to reduce EpC is associated with increased conduction velocity (CV, black to white points). The positive slope calculated from a linear fit of CV over the range of extracellular potassium (K^+^) from 4.56 to 7 mM is reduced as *W*_P_ decreases. The negative slope associated with sodium channel inactivation over the range of 9- to 10-mM K^+^ decreases to a greater extent with narrow *W*_P_, (b) Increasing Na^+^ by 10 mM (+10-mM Na^+^) decreases the positive slope of the CV-K^+^ relationship without substantively altering the negative slope or CV overall, (c) Reducing GJC by 50% (0.5 × GJC) slows CV for all values of K^+^ without changing the positive CV-K^+^ slope. However, 0.5 × GJC is associated with enhanced CV slowing during sodium channel inactivation measured between 9- and 10-mM K^+^ relative to the nominal condition in panel b. CV slowing was still the lowest with the narrowest cleft widths (0.5 × *W*_P_), (d) Reducing gNa by 50% (0.5 × gNa) not only reduces CV for all K^+^, but it also reduces both the positive and negative slopes of the CV-K^+^ relationship, without altering predictions that *W*_P_ associated with the slowest CV attenuates CV-dependent changes on K^+^

To explore potential mechanisms that may explain the experimental results above, the CV-K^+^ relationship was investigated in silico. The computational model used in Fig. 6 includes GJC and sodium channel localization at the ends of myocytes facing a shared and restricted extracellular cleft with a variable cleft resistance inversely proportional to perinexal width (*W*_P_). The family of curves in each panel of Fig. 6a–d represents narrow (0.5 × *W*_P_), nominal (1 × *W*_P_), and wide clefts (2 × *W*_P_) to demonstrate how modulating EpC alters the CV-K^+^ relationship.

The model predicts that progressively narrowing *W*_P_ (white to black curves), which enhances EpC, can decrease CV under conditions of robust GJC and EpC (Fig. 6a). Altering *W*_P_ does not dramatically change the positive slope of the linear portion of the CV-K^+^ curve estimated during supernormal conduction (4.56- through 7-mM K^+^). However, the negative CV-K^+^ slope estimated between 9- and 10-mM K^+^ is significantly reduced by narrowing *W*_P_. Specifically, the negative slope is −1.1 cm·mM/sec for the widest clefts (2 × W_P_) and −0.8 cm·mM/sec for the narrowest clefts (0.5 × W_P_). In short, the model predicts that narrow clefts can attenuate CV slowing during sodium channel inactivation due to hyperkalemia without notably altering supernormal conduction.

Elevating Na^+^ by 10 mM in this model can modestly increase CV, and narrowing *W*_P_ still slows CV. Interestingly, elevating Na^+^ decreases the positive slope of the linear portions of the CV-K^+^ relationship (Fig. 6b). In contrast, the negative slope associated with sodium channel inactivation at 10-mM K^+^ is not as dramatically affected by elevating Na^+^, and the values are similar to the negative slopes under the nominal case in Fig. 6a. Taken together with Fig. 6a, the model predicts that elevating Na^+^ can reduce the sensitivity of CV to changes in K^+^ during supernormal CV, but the negative slope is still predominantly determined by intercellular cleft width (*W*_P_) and therefore EpC. Importantly, elevating Na^+^ and reducing *W*_P_ attenuate the sensitivity of CV to K^+^ during supernormal conduction and sodium channel inactivation.

The CV response to reducing GJC by 50% (0.5 × GJC) can be found in Fig. 6c. As expected, reducing GJC slows CV, and narrowing *W*_P_ still slows CV. The model also suggests that inhibiting GJC does not substantively alter the CV-K^+^ relationship during supernormal conduction between 4.56- and 7-mM K^+^, but GJC inhibition can increase CV sensitivity to sodium channel inactivation as evidenced by larger negative slopes in Fig. 6c relative to 6a. Yet, the sensitivity of CV slowing during sodium channel inactivation is still the lowest under conditions of the narrowest cleft widths (0.5 × W_P_) and therefore elevated EpC.

When the peak conductance of the fast sodium current is inhibited by 50% (0.5 × gNa), CV slows dramatically as expected (Fig. 6d). Although narrowing *W*_P_ still slows CV, reducing *W*_P_ also reduces the positive and negative slopes of the CV-K^+^ relationship. In summary, the model predicts that enhancing EpC by narrowing *W*_P_ reduces CV sensitivity to loss of functional sodium channels induced by increasing sodium channel inactivation (10-mM K^+^) or reduced peak current (0.5 × gNa). In summary, computational models incorporating both EpC and GJC predict complex CV-K^+^ relationships with a consistent finding that narrowing *W*_P_ attenuates CV slowing during sodium channel loss of function induced by sodium channel inactivation or reduced peak sodium current.

## Discussion

The purpose of this study is to report how elevations in Na^+^ and/or Ca^2+^ modify the CV-K^+^ relationship and determine how these interventions modify the relationship when GJ coupling is reduced. We previously demonstrated that modification of Na^+^ and Ca^2+^ can alter CV in a variety of settings including genetically reduced Cx43 functional expression [13], in hearts exposed to the inflammatory cytokine TNFα [15], simulated metabolic ischemia [16], and global no-flow ischemia [26, 27]. To our knowledge, this is the first study to demonstrate that elevating Na^+^ and Ca^2+^ and pharmacologically inhibiting GJC will modulate the CV-K^+^ relationship nonlinearly.

### The relationship between conduction and K^+^

It is well established that the CV-K^+^ relationship is biphasic and can be modulated by other ions such as hydrogen (pH) [32, 52, 35]. In the range of hypo-, normo-, and mild-hyperkalemia, the relationship between CV and K^+^ is positively correlated, and supernormal conduction is the term coined to describe the observed increase in cardiac CV as K^+^ rises above normal plasma concentrations. Mechanistically, elevating extracellular K^+^ depolarizes the plasma membrane and reduces the difference between the RMP and the voltage gated sodium channel activation threshold [30, 44]. As a result, the net cellular charge accumulation required to reach the sodium channel activation threshold is reduced, sodium channel activation occurs earlier, and CV increases. It is equally well established that voltage gated sodium channel inactivation is related to membrane potential, as postulated most famously by Hodgkin and Huxley in 1952 [25, 24]. Therefore, as the RMP continues to rise consequent of increased extracellular K^+^, more sodium channels accumulate in the inactive state and the net sodium channel availability will decrease.

### Supernormal conduction

Our data demonstrate that CV increases when K^+^ is elevated between 4.6 and 6.4 mM with presumably normal GJC, and this is consistent with previous studies and mathematical models [32, 61]. Importantly, our data also suggest that supernormal conduction is relatively insensitive to the investigated changes of Na^+^ and Ca^2+^ used in this study. Of note, the relatively nonspecific GJC inhibitor CBX (30 μM) decreases CV for all K^+^ concentrations and abolishes the significant increase in supernormal CV when K^+^ is elevated from 4.6 to 6.4 mM. This could be due to an effect size below our resolution to detect as a result of increased CV variability introduced by the compound, or CBX actually decreases supernormal CV sensitivity.

Adding further complexity, the data presented herein are in apparent contrast with one of our previous studies in which CV decreased consequent of a modest elevation in K^+^ (from 4.1 to 6.1 mM) in conjunction with 155-mM Na^+^ and 1.8-mM Ca^2+^ in a murine model [18]. This may be due to the fact that there are underlying differences in normal plasma electrolyte concentrations between murine and guinea pig models [62]. The possibility that the CV-K^+^ relationship may be species dependent will require further investigation and may provide further insight into the complex regulation of cardiac CV.

### Conduction slowing due to sodium channel inactivation

As mentioned previously, K^+^-induced RMP depolarization will eventually impinge on steady-state sodium channel inactivation and decrease total sodium channel availability [61, 74]. In our study, elevating K^+^ from 4.6 to 10 mM slows CV for every Na^+^ and Ca^2+^ combination except one. Interestingly, and regardless of the presence of CBX, 155-mM Na^+^ and 2.00-mM Ca^2+^ is the only ionic combination in this study that is not associated with CV slowing at 10-mM K^+^. It is important to note that this lack of change in CV from 4.6 mM K^+^ may be due to experimental undersampling of the continuous CV-K^+^ relationship. There are at least four possible explanations for this finding. First, this could be a type II statistical error. However, the finding that CV does not change between 4.6- and 10-mM K^+^ only with the 155-mM Na^+^/2.00-mM Ca^2+^ perfusate is consistent with and without CBX. This increases our confidence that the resultis not a statistical error. Second, the ionic combination could minimize CV changes across the range of K^+^ values studied. Third, the mechanisms governing super-normal conduction and those governing conduction slowing due to sodium channel inactivation could be related but produce fundamentally different CV-K^+^ slopes. Lastly, the ionic combination could attenuate conduction slowing by right-shifting the K^+^ range that slows CV.

### Proposed mechanisms: cellular determinants of conduction

Divalent cations, such as Ca^2+^, inhibit *I*_K1_ [4]. Inhibition of *I*_K1_ should depolarize the RMP and therefore left shift the CV-K^+^ curve. If this were responsible for our experimental results, one would expect that 2.00-mM Ca^2+^ should significantly slow conduction more at 10-mM K^+^ relative to 1.25-mM Ca^2+^. We did not observe this. Specifically, CV slows similarly for 1.25- and 2.00-mM Ca^2+^ groups at 145-mM Na^+^. Furthermore, elevating Ca^2+^ actually attenuates conduction slowing at 155-mM Na^+^.

We also appreciate that Ca^2+^ is a potent secondary messenger and essential for enzymes including Ca^2+^/calmodulin-dependent protein kinase II (CaMKII) [65, 28, 72]. With regard to cardiac conduction, some have reported that increasing CaMKII activity can negatively shift sodium channel steady-state inactivation, thereby reducing peak *I*_Na_ [1, 3, 21]. Elevating Ca^2+^ and even decreasing Na^+^ should be associated with enhanced intracellular Ca^2+^ [7, 34], increased CaMKII activation, reduced *I*_Na_, and therefore conduction slowing. Once again, our results are not entirely consistent with the hypothesized effects of CaMKII activation. For example, one would expect to measure the greatest conduction slowing with solutions containing elevated Ca^2+^ and/or decreased Na^+^. Even though conduction slowing is observed with 145-mM Na^+^/1.25-mM Ca^2+^, conduction slows similarly to 145-mM Na^+^/2.00-mM Ca^2+^ and 155-mM Na^+^/1.25-mM Ca^2+^. Only 155-mM Na^+^/2.00-mM Ca^2+^ is not associated with significant conduction slowing at 10-mM K^+^, and this should be slower than conduction measured with 145-mM Na^+^/2.00-mM Ca^2+^, because reduced extracellular Na^+^ should also increase intracellular Ca^2+^ [34]. Importantly, the data do not exclude the importance of CaMKII post-translational modification of Nav1.5.

### Proposed mechanisms: intercellular coupling determinants of conduction

To our knowledge, this is the first study to evaluate the CV-K^+^ relationship consequent to GJ uncoupling. While the GJC inhibitor CBX has documented non-GJ-related off-target effects, it is a well-established model of pharmacologically induced GJC inhibition in cardiac preparations [20, 37, 10]. Regardless of the concentration of Na^+^ or Ca^2+^, CBX slowed conduction relative to preparations without CBX. Similar to preparations without CBX, the addition of CBX did not produce significant differences in the CV-K^+^ relationship with different perfusate combinations. Interestingly, the lack of observable conduction changes consequent of perturbations to Na^+^ or Ca^2+^ in this study at 4.6-mM K^+^ is in contrast to individual ion associated changes in conduction observed in the aforementioned murine model of genetically reduced Cx43 [18]. However, the combination of enhanced Na^+^ and Ca^2+^ does attenuate CBX-induced CV slowing in guinea pig consistent with findings in mice with genetically downregulated Cx43.

The fact that the findings with pharmacologically induced GJC inhibition in guinea pig does not correlate one to one with genetically reduced functional GJC in mouse warrants additional investigation. Factors to consider include the off-target effects of CBX, the unknown degree of GJC inhibition elicited by CBX, and the fact that genetically manipulated animal models of protein functional expression are associated with off-target protein remodeling [64, 55, 29, 49]. Still, the conclusion that ionic modulation of cardiac conduction can be GJ-dependent is consistent across species with different interventions to induce GJ uncoupling. Understanding species differences, particularly as mice have different plasma electrolyte concentrations from human as noted above, is important since individual variations in extracellular ion homeostasis may underlie subtle variations in phenotype [39].

The computational model is important to inform our understanding of how GJC and enhanced EpC (by reduced *W*_P_) modulate conduction under the many conditions experimentally explored in this study. We first note that the model is tuned to study CV in the range of significant EpC self-attenuation in the presence of robust GJC. Briefly, self-attenuation is proposed to work in two ways. First, activation of voltage gated sodium channels facing narrow extracellular nanodomains will decrease the junctional extracellular potential (Φ_J_) by charge withdrawal from the nanodomain and raise the transmembrane potential (*V*_m_) of apposing membranes, where [*V*_m_ = (Φ_i_ − Φ_J_)] and Φ_i_ represents the intracellular potential. Secondly, sodium withdrawal from the nanodomain also decreases extracellular Na^+^ and, by the Nernst equation, will decrease the sodium equilibrium potential (*E*_Na_) and therefore the driving force for ion entry into the cell [Driving Force = (*V*_m_ – *E*_Na_)]. As both the extracellular potential (Φ_J_) and *E*_Na_ rapidly decrease, the driving force for Na^+^ through sodium channels approaches zero more rapidly and hence the term “self-attenuation.” Under conditions of robust GJC, the intracellular potential (Φ_i_) also rises more rapidly due to current through GJs and causes *V*_m_ to increase rapidly. Thus, with both elevated GJC and EpC, all three parameters that define the driving force [Φ_i_ − Φ_J_ − *E*_Na_] for sodium channels change concurrently and reduce peak *I*_Na_ at the intercalated disc, while simultaneously increasing the rate of voltage gated sodium channel opening as a result of rapid *V*_m_ changes. In short, the relationship between EpC, GJC, and CV is complex because both mechanisms are important for determining the rate of action potential propagation, while simultaneously limiting peak *I*_Na_.

As a result of these complementary mechanisms, the relationship between CV and the cleft width between neighboring myocytes is biphasic [40, 41]. Models predict that CV will increase when cleft separation between myocytes increases but only when clefts are relatively narrow. In this range, widening clefts reduces self-attenuation and increases CV. As clefts continue to widen the role of EpC action potential transmission is reduced, and CV becomes more dependent on GJC. The slower intracellular charge transfer through GJC still maintains robust cardiac conduction, but CV decreases as EpC is removed from the process of intracellular communication. This is important, because models of GJC and EpC should consider the assumptions of EpC and GJC when extrapolating to potential mechanisms. Case in point, in Supplemental Figure 3, GJC is reduced by 98% to increase CV dependence on EpC. Under these conditions, narrowing cleft width now increases CV in contrast to Fig. 6a where GJC is robust. Under conditions of almost no GJC, narrowing cleft width increases the sensitivity of the CV-K^+^ relationship to *W*_P_ in a manner opposite to what is found in Fig. 6a. Interestingly, the model in Fig. 6 and Supplemental Figure 3 suggests that when EpC is present, the cleft widths associated with slowest CV attenuate supernormal CV and CV slowing consequent to sodium channel loss of function.

Furthermore, the model presented in Fig. 6 replicates key experimental conditions and outcomes, suggesting that elevating Na^+^ and reducing *W*_P_ can attenuate the positive and negative slopes of the CV-K^+^ relationship, but the model is not precisely tuned to replicate the experimental finding that 10-mM K^+^ slows CV below values obtained at 4.6-mM K^+^ in experiments. The model also predicts that altering *W*_P_ can attenuate the positive CV-K^+^ slope during supernormal CV, but this was not observed experimentally. However, the model may have explored changes in *W*_P_ greater than those experimentally induced, CV changes could be below our resolution to experimentally detect, or our model assumptions require further tuning in a complex and emergent multiparametric space. This assertion is not just conjecture. Computational models have long predicted that altering the distribution of sodium channels in the lateral membrane and cleft to include electric field and EpC dramatically alters myocardial electrophysiology to explain contentious issues including the relationship between CV and GJC [38, 48, 40, 68, 70, 18, 73, 22, 16, 27], CV and sodium channel loss of function [66, 70, 67], and APD and sodium channel gain of function [19, 50, 51]. Importantly, the experimental data and computational models suggest that both EpC and GJC are sufficiently robust in the experimental setup used in this study, such that small changes in *W*_P_ may either modestly increase CV or not change it significantly as reported above. The finding that CV slows to a similar degree at 10-mM K^+^ with and without GJC inhibition is also worth noting, as the model suggests CV slowing should be enhanced with GJC, but we do not know (1) if the expected CV change was below our resolution to detect, (2) the degree to which 30-μM CBX inhibits GJC, or (3) whether previously reported *W*_P_ narrowing with CBX [11] reduces the negative slope of the CV-K^+^ relationship during sodium channel inactivation. Regardless, the experimental data and computational models are complementary. The solution associated with the strongest EpC (155-mM Na^+^/2.00-mM Ca^2+^) is not associated with conduction slowing at 10-mM K^+^. In other words, this study supports a hypothesis that enhanced EpC attenuates CV slowing secondary to functional loss of voltage gated sodium channels.

### Asystole in the presence of severe hyperkalemia

In the presence of 145-mM Na^+^, with normal GJC, the majority of hearts became asystolic during 10-mM K^+^ perfusion. This is likely due to a reduced sodium channel availability consequent to extracellular K^+^-induced membrane depolarization. In contrast, a significantly higher number of hearts remained in intrinsic rhythm during 10-mM K^+^ perfusion in the presence of 155-mM Na^+^. The mechanism by which this occurs is unknown, since asystole may be caused by altered automaticity, a lack of excitability, or a failure for the excitable signal to propagate into the myocardium.

Inhibiting GJs with CBX significantly decreases the incidence of asystole in hearts perfused with 145-mM Na^+^ and 10-mM K^+^. This is an unexpected finding as precedent literature provides evidence for a necessary role of robust GJC in coordinated nodal tissue excitability [9, 46, 47]. An important caveat of our experimental condition however is that the atria, and thereby the sino-atrial node, are removed from the preparation. Previous literature also provides that limiting current sink, as would happen in the context of uncoupled GJs, lowers the source charge required for excitation [60]. Per this line of thought, uncoupling GJs may facilitate entrained or propagated autorhythmic coordination in the context of otherwise lowered cellular excitability (severe hyperkalemia). The precise mechanism of how GJ uncoupling maintains intrinsic rhythm in the context of severe hyperkalemia warrants further investigation.

### Perspective on isolated heart experiments

These results have important implications for preclinical cardiovascular research, particularly in the context of isolated organ experiments, when considering how laboratory perfusates are chosen. The level of variation in perfusate composition between laboratories is significant. Common differences in perfusate compositions include modifications to ubiquitous electrolytes such as Na^+^, Ca^2+^, K^+^, Mg^2+^, and Cl^−^, buffering agents, electro-mechanical uncouplers, pH, and addition of any proteins or fatty acids. The results of this manuscript, along with many of our previous studies, highlight the impact that modest changes in electrolyte composition can have on cardiac function in the Langendorff-perfused ex vivo heart and may help to explain some experimental discrepancies found in the literature.

### Clinical implications

Clinically, hyperkalemia is associated with diseases such as ischemia, renal failure, and HIV; medications such as ACE inhibitors, penicillin, and heparin; and acute injury such as crush injury and burns [53, 74]. Severe hyperkalemia, if left untreated, can result in sudden cardiac death. One mechanism by which hyperkalemia can lead to sudden cardiac death is by elevating the RMP and effectively reducing sodium channel availability.

While our present study investigates the consequence of variations in Na^+^ and Ca^2+^ in the context of hyperkalemia, the results have broader implications for diseases associated with decreased sodium channel functional expression or diseases aggravated by increased extracellular K^+^. For example, hyperkalemia-induced steady-state inactivation may mimic the loss of *I*_Na_ associated with multiple diseases such as some congenital forms of Brugada syndrome and Duchenne muscular dystrophy [2, 54, 36]. In fact, the computational modeling in Fig. 6 suggests that narrowing *W*_P_ attenuates CV slowing during sodium channel loss of function either due to sodium channel inactivation (10-mM K^+^) or reduced channel unitary conductance (0.5 × gNa). The results are timely as clinical case reports suggest that both Brugada syndrome and Brugada phenocopies may be either unmasked or induced, respectively, by hyperkalemia [59]. The finding that conduction slowing is greatest secondary to hyperkalemic sodium channel inactivation under conditions of reduced EpC is consistent with similar findings where pharmacologic sodium channel inhibition is exacerbated by reduced EpC [71, 67]. This study, in the context of previous findings, supports a hypothesis that Brugada syndrome is modulated by EpC. In fact, a very recent and independent computational study supports this hypothesis by demonstrating how EpC can modulate conduction and phase 2 reentry in Brugada syndrome [67]. Thus, our study supports a growing body of evidence that EpC may be an effective target to treat cardiac diseases associated with a reduction in myocardial sodium current density. Though, further studies are needed to determine whether such ionic changes may indeed modulate conduction deficits in the context of diseases with reduced functional sodium channel expression in a clinical setting.

## Conclusion

In the present study, we provide evidence that altering Na^+^ and Ca^2+^ will modify the well-established CV-K^+^ relationship. These results again demonstrate the importance of perfusate composition in studying cardiac function. At the bench, the respective changes in electrolyte concentrations may seem small but can have profound impacts on cardiac function and may even explain disparate outcomes within the field [13, 16, 34].

Considering the frequency of presentation and pernicious nature of clinical hyperkalemia, it is important that we further understand the influence of electrolytic imbalances on the CV-K^+^ relationship. While our results do not offer an immediate therapeutic option, they do suggest that the combined elevation of Na^+^ and Ca^2+^ could preserve CV in the context of severe hyperkalemia and/or sodium channel loss of function and may have the potential to reduce arrhythmogenic conduction slowing and block.

## Supplementary Information

ESM 1 (DOCX 69 kb)

## Data Availability

The datasets generated and/or analyzed during the current study are available from the corresponding author on reasonable request.
