# Supplementary material for: The conduction velocity-potassium relationship in the heart is modulated by sodium and calcium
Source: Pflugers Arch. 2021 Mar 4;473(3):557–71. doi: 10.1007/s00424-021-02537-y (PMC7940307; doi:10.1007/s00424-021-02537-y)
Supplement: Supplementary file 1 — (DOCX 69 kb) [file 424_2021_2537_MOESM1_ESM.docx]

**Supplemental Information – Statistical Modeling**

**CV-K^+^ as a function of perfusate Na^+^ and Ca^2+^**

A linear model was fit to determine if the change in CV due to elevation of K^+^ differed across perfusate groups (varied Na^+^ and Ca^2+^). Models for CV_L_ and CV_T_ were fit independently. In both cases, 145mM Na^+^ / 1.25mM Ca^2+^ was chosen as the baseline condition and a separate effect fit was generated for each of the four Na^+^/Ca^2+^ concentration variations. These data are presented in Supplemental Figure 1, Supplemental Table 1, and Supplemental Table 2.


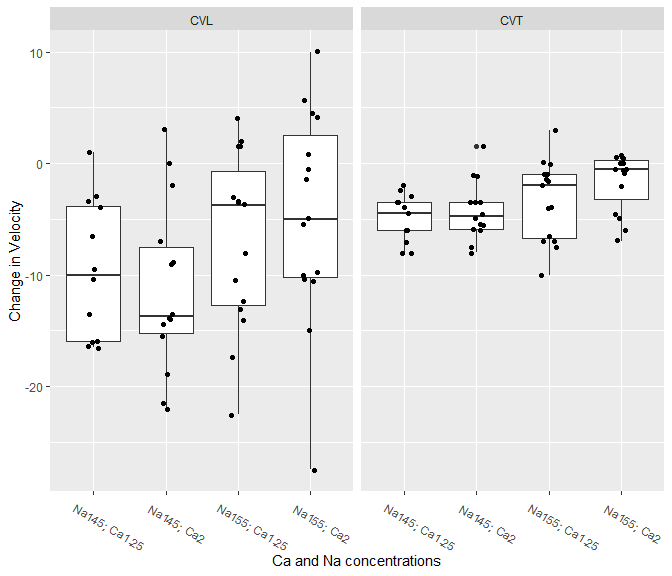


**Supplemental Figure 1**. Boxplots of change in CV_L_ and CV_T_ between K^+^=4.6mM and K^+^=10.0mM within perfusate groups.

Supplemental Table 1: Model coefficients for changes in CV_T_ from 4.6mM K^+^ to 10.0mM K^+^

|  | Estimate | SD | T | p |
| --- | --- | --- | --- | --- |
| Baseline (Na 145; Ca 1.25) | -4.923 | 0.778 | -6.332 | 0.000 |
| Na 145; Ca 2 | 0.691 | 1.080 | 0.640 | 0.525 |
| Na 155; Ca 1.25 | 1.590 | 1.062 | 1.496 | 0.140 |
| Na 155; Ca 2 | 3.273 | 1.062 | 3.081 | 0.003 |

Supplemental Table 2: Model coefficients for changes in CV_L_ from 4.6mM K^+^ to 10.0mM K^+^

|  | Estimate | SD | T | p |
| --- | --- | --- | --- | --- |
| Baseline (Na 145; Ca 1.25) | -9.542 | 2.324 | -4.105 | 0.000 |
| Na 145; Ca 2 | -1.744 | 3.168 | -0.551 | 0.584 |
| Na 155; Ca 1.25 | 2.692 | 3.119 | 0.863 | 0.392 |
| Na 155; Ca 2 | 4.842 | 3.119 | 1.553 | 0.127 |

The results in Supplemental Table 1 show that, at the 0.05 level, none of the CV_L_ changes for any of the perfusate groups differ from that of the baseline concentration. Supplemental Table 2 shows that when both Na^+^ and Ca^2+^ are elevated, the CV_T_ change between 4.6mM K^+^ and 10.0mM K^+^ is significantly different from baseline. If either Na^+^ or Ca^2+^ are elevated independently the difference in CV_T_ is not significant. For the baseline group, the average CV_T_ is lower when K^+^ = 10mM versus when K^+^ = 4.6mM by 4.923 cm/s. When both Na^+^ and Ca^2+^ are elevated CV_T_ is lower by 1.65 cm/s.

**Parabolic Fits**

Mixed effects models (doi: 10.18637/jss.v067.i01) were fit to estimate CV as a quadratic function of K^+^. A parabola was fit for each Na^+^ and Ca^2+^ concentration simultaneously and random intercepts were included to account for correlation within the hearts. The fitted parabolas for CV_L_ and CV_T_­ appear in Supplemental Figure 2; their corresponding parabolic coefficients are included in Supplemental Table 3 and Supplemental Table 4.


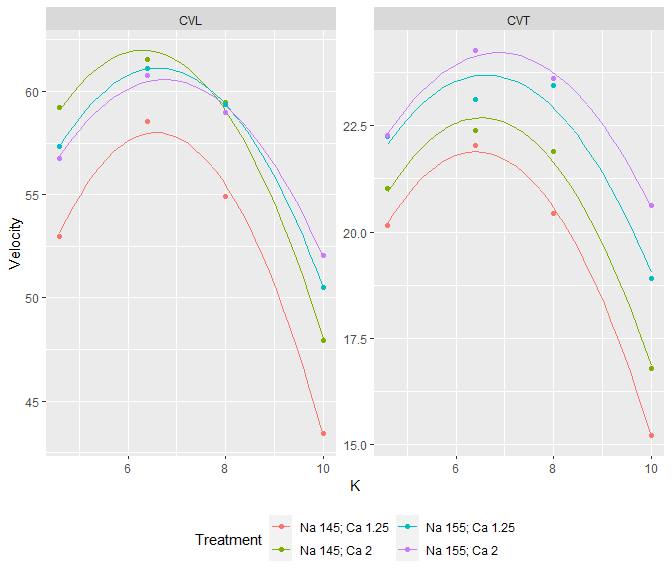


**Supplemental Figure 2.** Quadratic fits of CV as a function of K^+^ for each Na^+^/Ca^2+^ combination. The points on the curves indicate the mean values for each K^+^ as obtained experimentally (Figure 3).

The parabolic fit of the 145mM Na^+^ / 1.25mM Ca^2+^ perfusate group was chosen as baseline for all comparisons. None of the values of the parameters for the other ion concentrations differed from the baseline values at the 0.05 level. In the remainder of the analysis, we highlight trends and patterns in an exploratory way, noting none of the fits are statistically different from one another.

The coefficients of the parabolic models for CV_L_ are given in Table 3 and for CV_T_ in Table 4. For each parabola, the apex gives the K^+^ at which the velocity is highest. It is also the point at which the direction of the relationship between K^+^ and CV changes. As K^+^ increases, CV increases as long as K^+^ is below this value and decreases when K^+^ is above this value.

The curvature is the absolute value of the second derivative of the function and is a measure of how quickly the function falls off. Large curvatures indicate that CV decreases rapidly as K^+^ increases past the apex. Curvatures close to zero indicate that velocity decreases slowly as K^+^ increases past the apex.

Table 3: Parabolic coefficients as well as the apex and curvature for the CV_L_ parabolas

|  | Constant | Linear | Quadratic | Apex | Curvature |
| --- | --- | --- | --- | --- | --- |
| Baseline (Na 145; Ca 1.25) | 3.819 | 16.493 | -1.255 | 6.572 | 2.510 |
| Na 145; Ca 2 | 21.666 | 12.811 | -1.017 | 6.297 | 2.035 |
| Na 155; Ca 1.25 | 20.363 | 12.323 | -0.931 | 6.619 | 1.862 |
| Na 155; Ca 2 | 23.833 | 10.901 | -0.809 | 6.740 | 1.617 |

Table 4: Parabolic coefficients as well as the apex and curvature for the CV_T_ parabolas.

|  | Constant | Linear | Quadratic | Apex | Curvature |
| --- | --- | --- | --- | --- | --- |
| Baseline (Na 145; Ca 1.25) | 0.649 | 6.633 | -0.518 | 6.404 | 1.036 |
| Na 145; Ca 2 | 2.371 | 6.237 | -0.479 | 6.513 | 0.958 |
| Na 155; Ca 1.25 | 6.069 | 5.332 | -0.403 | 6.609 | 0.807 |
| Na 155; Ca 2 | 6.791 | 5.073 | -0.369 | 6.866 | 0.739 |

Velocity will be higher for large values of K^+^ either when the apex is large, the curvature is low, or both. In both tables, the apex is smallest and the curvature largest when Na^+^ and Ca^2+^ are in low concentrations, while the apex is largest and the curvature is smallest when both ions are in high concentrations. This is consistent with the findings above which showed, at least for CV_T_, that large Na^+^ and Ca^2+^ concentrations tend to result in higher velocities when the K^+^ concentrations were high.

**Simulations of the CV-K^+^ relationship under conditions of very low Gap Junction Coupling (GJC)**

In contrast to Figure 6 in the main document, the CV-K^+^ relationship is plotted for 98% reduced GJC (0.02xGJC). Under these model conditions. Widening perinexal width (W_P_) now decreases CV in contrast to Figure 6. At the same time, expanding W_P_ reduces the positive slope of the CV-K^+^ relationship for K^+^ between 4.56 and 7mM and reduces the negative slope of the CV-K^+^ relationship for K^+^ between 9 and 10mM. Considered together with Figure 6 in the manuscript, the model suggests that slowing CV by modulating W_P_ regardless of GJC, will reduce CV sensitivity to K^+^.


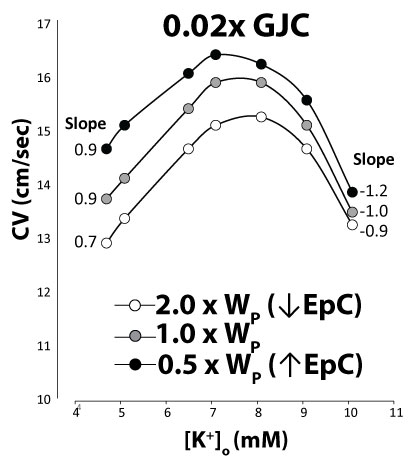


**Supplemental Figure 3.** Computational predictions of modulating perinexal width (W_P_), extracellular sodium concentrations (Na^+^), gap junctional coupling (GJC), and the fast sodium channel conductance (gNa). During 98% reduced GJC (0.02xGJC), increasing W_P_ slows CV as a result of reduced ephaptic coupling (EpC). W_P_ associated with the slowest CV values for all K^+^ have the shallowest positive and negative slopes consistent with Figure 6A in the main body of the manuscript.
